# Intraoperative Ultrasound as a Decision-Making Tool in Modern Gynecologic Oncology

**DOI:** 10.3390/jpm15070296

**Published:** 2025-07-08

**Authors:** Mohamed Lakany, Amana Sharif, Moiad Alazzam, Catherine Howell, Sian Mitchell, Christina Pappa, Dana Shibli, Lisa Story, Ahmad Sayasneh

**Affiliations:** 1 Gynaecological Oncology Department, Guy’s & St Thomas’ Hospital NHS Foundation Trust, London SE1 7EH, UK; mohamed.ellakany@nhs.net (M.L.); amana.sharif@gstt.nhs.uk (A.S.); sian.mitchell@gstt.nhs.uk (S.M.); lisa.story@kcl.ac.uk (L.S.); ahmad.sayasneh@gstt.nhs.uk (A.S.); 2 Gynaecological Oncology Department, Oxford University Teaching Hospitals NHS Foundation Trust, Headington, Oxford OX3 7LE, UK; christina.pappa@ouh.nhs.uk; 3 GynaeFellow Educational Collaborative, Headington, Oxford OX3 7LE, UK; catherine.howell1@nhs.net (C.H.); shiblidana76@gmail.com (D.S.); 4 Calderdale and Huddersfield NHS Foundation Trust, Women’s Health, Halifax HX3 0PW, UK; 5 Jordan University Hospital, Amman 11942, Jordan; 6 King’s College, Strand Campus, London WC2R 2LS, UK

**Keywords:** intraoperative ultrasound, gynecologic oncology, surgical precision, cytoreduction, fertility preservation, lymph node assessment, image-guided surgery, ovarian cancer

## Abstract

**Background:** Intraoperative ultrasound (IOUS) is revolutionizing gynecologic oncology surgery by overcoming the limitations of traditional imaging and intraoperative assessment. Its real-time, high-resolution capabilities address critical needs in tumor localization, fertility preservation, refined intraoperative decisions, and complete cytoreduction. **Methods:** We reviewed clinical studies (1998–2024) evaluating IOUS applications, analyzing data on detection accuracy, surgical outcomes, and implementation challenges from peer-reviewed literature and institutional experiences. **Results:** IOUS demonstrates 88–93% sensitivity for subcentimeter metastases, refining surgical decisions in 25–40% of cases. Key outcomes include increased complete resection rates (68% to 87%), a 38% reduction in unnecessary lymphadenectomies, and successful fertility preservation in 92% of cases. Limitations include learning curves, 12% false-negative rate for micrometastases, and significant capital investment cost barriers. **Conclusions:** IOUS represents a transformative advance in precision surgery, improving both oncologic outcomes and quality of life. While standardization and accessibility challenges remain, ongoing technological innovations promise to solidify its role as a surgical standard.

## 1. Introduction

The development of diagnostic ultrasound by Ian Donald and Tom Brown in 1958 marked a watershed moment in medical imaging, particularly for gynecologic and obstetric applications [1]. Over subsequent decades, ultrasound became the cornerstone of pelvic imaging due to its unparalleled safety profile (non-ionizing radiation), cost-effectiveness, and real-time diagnostic capabilities [2]. It remains indispensable for evaluating early pregnancy complications, characterizing ovarian pathologies, and diagnosing uterine abnormalities in patients presenting with bleeding disorders or infertility [2,3].

Despite its diagnostic ubiquity, the adoption of intraoperative ultrasound (IOUS) in gynecologic surgery has progressed slower than in other surgical disciplines. Urology pioneered IOUS in the 1960s [3], and today it is integral to renal tumor resection, where it enables nephron-sparing precision [4,5,6]. Similarly, hepatobiliary and neurosurgical specialties have fully embraced IOUS as a critical intraoperative guidance tool [7]. In contrast, gynecologic oncology has been slower to incorporate this technology, despite mounting evidence of its potential to address unique surgical challenges in the female pelvis [8,9,10,11,12].

The management of gynecologic malignancies presents distinct complexities where millimeter-level precision determines oncologic outcomes. Complete cytoreduction, fertility preservation, and nerve-sparing techniques demand real-time anatomic intelligence that conventional preoperative imaging cannot provide [13,14,15]. Traditional reliance on preoperative MRI/CT and intraoperative palpation has been proven particularly inadequate when dealing with small (<1 cm) or deeply embedded lesions, non-palpable lymph nodes, or distorted anatomy from prior surgeries or previous radiotherapy [16,17,18]. These limitations directly impact critical outcomes: residual disease following debulking surgery, unnecessary radical procedures in early-stage disease, and iatrogenic injury to vital structures.

IOUS emerges as a transformative solution to these challenges by providing dynamic, high-resolution visualization during the surgical decision-making process. Its applications span the full spectrum of gynecologic oncology:

- Localizing occult tumors in fertility-sparing surgery [19,20,21]
- Guiding complete resection of deep infiltrating endometriosis [22]
- Identifying metastatic lymph nodes missed by preoperative imaging [23,24,25]
- Optimizing cytoreduction in advanced ovarian cancer [26,27,28]

The technology’s value is magnified in minimally invasive approaches, where the loss of tactile feedback is compensated by enhanced visual information [29,30]. Recent studies demonstrate that IOUS can change intraoperative decision-making in 25–40% of cases, preventing unnecessary radical procedures while ensuring complete tumor resection [31,32,33].

This narrative review examines the evolving role of IOUS in gynecologic oncology, synthesizing current evidence across four key domains:

1. Fertility preservation in borderline and early-stage malignancies
2. Precise lymph node assessment and staging
3. Optimization of cytoreductive surgery
4. Integration with minimally invasive platforms

We analyze the technical aspects of IOUS application, present clinical outcome data, and address persistent barriers to adoption. As personalized medicine reshapes oncologic care, IOUS stands poised to become an indispensable tool for tailoring surgical strategies to individual patient anatomy and disease distribution—fulfilling the promise of true precision surgery in gynecologic oncology.

### High-Resolution Intraoperative Ultrasound Systems in Gynecologic Oncology

Modern high-resolution ultrasound systems have become indispensable tools for intra-abdominal applications in gynecologic oncology. Leading platforms, including the bk5000 (BK Medical), GE LOGIQ E9, Aloka ProSound F75, Samsung RS85, and Philips EPIQ 7, offer a versatile array of transducers, such as convex, laparoscopic, and linear probes, compatible with open, laparoscopic, and robotic-assisted surgical approaches. These systems deliver high-frequency imaging (up to 18 MHz in select linear probes) and incorporate advanced modalities, including Doppler imaging, elastography, contrast-enhanced ultrasound (CEUS), and fusion imaging, which enhance intraoperative lesion characterization and vascular mapping [5].

The bk5000 is particularly optimized for surgical settings, featuring sterilizable laparoscopic probes and smart needle-tracking technology, making it a preferred choice in robotic gynecologic and colorectal oncology. Similarly, the GE LOGIQ E9 and Philips EPIQ 7 provide deep tissue penetration and real-time navigation, critical for complex tumor resections [7].

## 2. Materials and Methods

This narrative review synthesizes evidence from clinical trials, cohort studies, and case series identified in PubMed/MEDLINE searches between 1998 and 2023. We prioritized studies reporting quantitative outcomes on IOUS accuracy, surgical decision impact, and patient survival. Data extraction focused on study design, patient characteristics, surgical techniques, and comparative outcomes versus conventional methods. Inclusion criteria encompassed English-language publications with ≥10 cases and outcome measures. Expert consensus statements and society guidelines were incorporated where available.

## 3. Results and Discussion

- *Preoperative adhesion mapping with intraoperative ultrasound for safe surgical access*

Laparoscopic entry carries inherent risks, with vascular, visceral, and urologic injuries accounting for approximately 50% of major laparoscopic complications; the majority occurring during initial abdominal access [16]. Adhesion formation, caused by prior surgical history and incision type, further elevates this risk. Umbilical adhesions are present in up to 50% of patients following midline laparotomy and 23% following Pfannenstiel incisions [17]. In obese patients, where laparoscopy is preferred due to its association with reduced postoperative morbidity, anatomical landmark identification becomes particularly challenging. Elevated BMI (>35) correlates with increased entry attempts and difficulty in visualizing critical structures (e.g., inferior epigastric vessels), compounding the risk of iatrogenic injury [18].

**Figure 1:**
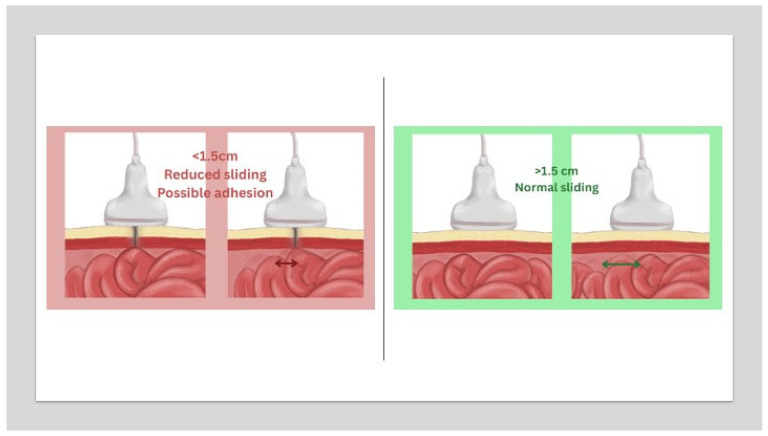
Visceral sliding (dynamic movement of visceral organs (such as the bowel or abdominal structures) against each other or the abdominal wall during respiration. Normal Finding: Smooth, gliding motion indicates the absence of adhesions or inflammation. Abnormal Finding: Reduced or absent sliding suggests pathology e.g., adhesions (courtesy of https://gynaefellow.com/).

Intraoperative ultrasound (IOUS) addresses these challenges through real-time, dynamic assessment of abdominal wall architecture. The visceral slide test; a validated, non-invasive technique, demonstrates high reliability in detecting periumbilical adhesions [19] (Figure 1).

The test can be performed using abdominal ultrasound just prior to entry to assess for any adhesions in patients of higher risk such as those with previous surgery and of increased BMI. A curve or linear ultrasound scan probe can be used to assess the bowel hyperechogenic content sliding below the abdominal wall with respiratory movement while asking the anesthetist to facilitate deep respiration. If the bowel slides freely more than 1.5 to 2 cm in relation to the abdominal wall during respiration, this is considered normal visceral sliding, suggesting no adhesions. Reduced or absent sliding (<1.5 cm) between the bowel and the abdominal wall suggests possible adhesions (Figure 1).

- *Borderline ovarian tumors; a paradigm shift*

Borderline ovarian tumors (BOTs) represent a unique diagnostic and therapeutic challenge in gynecologic oncology. With an incidence of 4.8 per 100,000 women, these epithelial neoplasms of low malignant potential account for 10–15% of all epithelial ovarian cancers, predominantly affecting women in their reproductive prime (30–50 years) [20,21]. While their indolent nature is reflected in 10-year survival rates exceeding 90%, these tumors occupy a precarious position between benign and malignant disease; a clinical grey zone where optimal management requires meticulous balancing of oncologic radicality while preserving the reproductive potential [22]. The surgical management of BOTs has traditionally been constrained by a fundamental dilemma: the conflict between radical excision and fertility preservation. Conservative, ovary-sparing approaches carry a two- to four-fold increased risk of recurrence compared to radical surgery [8], while oophorectomy represents an irrevocable compromise of reproductive potential. This tension is particularly acute in cases of recurrent disease, where lesions frequently present as small, non-palpable nodules that evade visual detection during conventional laparoscopy [23]. The characteristic sonographic features of serous BOTs—unilocular-solid cysts (79%) with irregular papillary projections (89%) [9]—often become invisible to the surgeon’s eye once embedded in ovarian parenchyma.

Modern ultrasound technology has achieved remarkable sensitivity in detecting these lesions preoperatively, with the capacity to identify tumors as small as 8–10 mm [8,9]. However, this diagnostic precision has historically been lost at the critical surgical moment, forcing surgeons to choose between radical excision of entire ovaries or blind cystectomy attempts that risk incomplete resection, iatrogenic rupture, or unnecessary sacrifice of healthy ovarian tissue [23]. The consequences of these limitations are particularly profound for nulliparous patients, who comprised 86% of cases in recent studies [10].

The advent of intraoperative ultrasound (IOUS) guidance represents a transformative solution to this clinical impasse. The technique, as pioneered by innovative surgical teams [10,24,25], integrates real-time sonographic visualization with minimally invasive surgery through a sophisticated multi-step approach:

1. Pelvic saline infusion (500 mL) creates an acoustic window for enhanced ultrasound transmission.
2. Transvaginal or laparoscopic probes provide multiplanar tumor localization.
3. Laparoscopic instruments mark lesion boundaries under dual visual-sonographic guidance.
4. Diathermy delineates precise resection margins before ultrasound-monitored excision.

The approach has been validated through a number of clinical studies. In a case series of seven patients (median age 35 years) with recurrent, laparoscopically occult sBOTs (median size 18 mm), IOUS-guided resection achieved 100% complete excision with no intraoperative complications [10]. Subsequent validation studies at high-volume centers reproduced these outcomes, with particular success in nulliparous patients [24,25]. The case reported by Mascilini et al. [9] exemplifies the technique’s precision—a 16 mm recurrent lesion, invisible to standard laparoscopy, was completely resected with ovarian preservation, confirmed by frozen section analysis.

**Figure 2:**
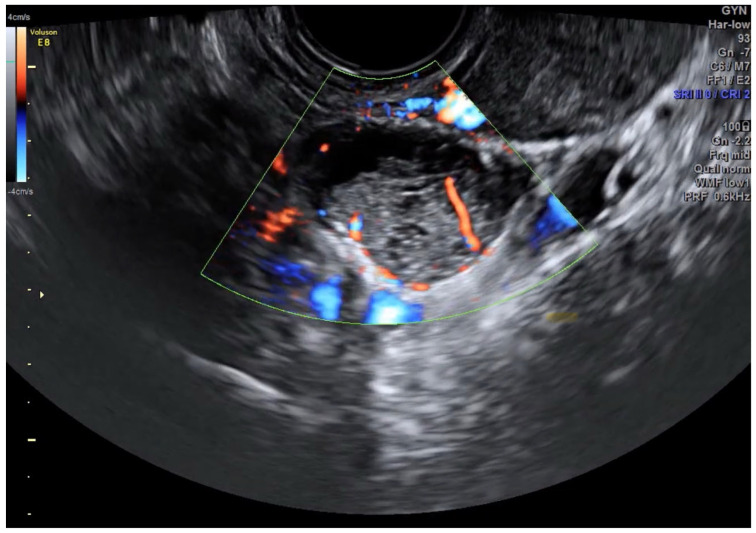
Identifying a less than 2 cm recurrent serous BOT intraoperatively (courtesy of A. Sayasneh).

Beyond technical success, IOUS addresses the fundamental philosophical challenge in BOT management: the reconciliation of oncologic and reproductive priorities. By making the invisible visible, it transforms surgical decision-making from a binary choice between radicality and conservation to a nuanced, patient-specific strategy. The technique’s ability to detect and guide resection of subvisual lesions ≤ 2 cm [10] represents a paradigm shift in our approach to fertility-sparing oncology (Figure 2).

As fertility-sparing approaches gain prominence among reproductive-age patients with borderline ovarian tumors (BOTs), intraoperative ultrasound (IOUS) has transitioned from an adjunct tool to a critical component of surgical decision-making. By enabling real-time delineation of tumor margins and stromal involvement, IOUS facilitates ovarian conservation without compromising oncologic principles addressing both the biologic and psychosocial dimensions of care.

Current evidence suggests that IOUS enhances the precision of cystectomy and stromal evaluation, potentially reducing the need for repeated interventions while maintaining low recurrence rates. Looking ahead, technological advancements such as contrast-enhanced ultrasound (CEUS) and AI-driven image analysis may further optimize detection of microscopic disease, offering a pathway toward true precision surgery in gynecologic oncology.

- *IOUS in gynecological oncology lymph node assessment and staging*

Nodal evaluation remains a cornerstone, yet a persistent challenge, in the surgical management of gynecologic malignancies. The current paradigm exists in tension between the oncologic necessity of precise staging and the significant morbidity linked to a systematic lymphadenectomy. This controversy manifests distinctly across disease sites: in borderline ovarian tumors (BOTs), where nodal involvement is rare (<5%) but portends potential occult invasive carcinoma necessitating therapeutic escalation [11], and in endometrial cancer, where the survival benefit of routine para-aortic lymphadenectomy for apparent early-stage disease remains contested [15].

Conventional cross-sectional imaging (CT/MRI) exhibits well-documented limitations in nodal assessment, with sensitivity to metastatic detection frequently ≤60% [27,28,29]. These modalities demonstrate dual shortcomings: inadequate resolution for micrometastases and limited specificity in distinguishing malignant infiltration from reactive hyperplasia.

Intraoperative ultrasound (IOUS) addresses these gaps through real-time, high-resolution nodal interrogation, synergizing the advantages of dynamic surgical assessment with advanced sonographic capabilities. Its diagnostic precision derives from multiparametric analysis of:

- Morphology (cortical thickening, spherical index, hilar integrity)
- Echostructure (heterogeneity, microcalcifications)
- Vascular dynamics (hilar vs. peripheral perfusion patterns)
- Biomechanical properties (strain elastography-derived stiffness ratios)

This integrated approach enables discriminative analysis of nodal basins, potentially refining intraoperative decision-making while mitigating the overtreatment inherent to prophylactic lymphadenectomy.

This diagnostic value of IOUS was powerfully demonstrated in the case reported by De Blasis et al. [11], in which IOUS identified a precaval lymph node with malignant features (irregular borders, lost hilum, microcalcifications) that was subsequently confirmed as metastatic low-grade serous carcinoma. This critical intraoperative finding prompted comprehensive para-aortic lymphadenectomy and subsequent adjuvant therapy; a decision that would have been omitted with conventional surgical inspection alone.

The Musashino Red Cross Hospital study [12,26] provided evidence for IOUS superiority over conventional MRI and CT scan staging for endometrial cancer. Their decade-long experience with 91 patients demonstrated IOUS provided higher sensitivity over CT in detecting para-aortic nodal metastases, particularly for lesions < 1 cm. The IOUS proved to be highly valuable in identifying “interval nodes”, nodes that are located between standard dissection boundaries, which might otherwise be missed. Importantly, the study highlighted IOUS’s practical advantages: rapid acquisition time (<5 min per nodal station), reasonable learning curve for trained sonographers, and significant cost savings compared to frozen section analysis.

**Figure 3:**
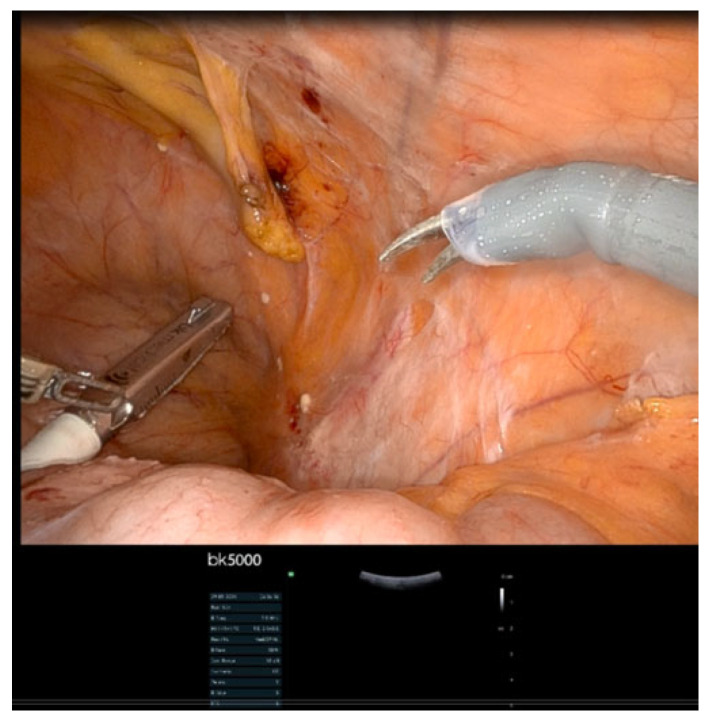
Plastered sidewall and distorted anatomy with thick peritoneum after multiple surgeries and many lines of treatments (immune and chemotherapy). Introducing the robotic BK 5000 ultrasound scan probe into the pelvis through the 12 mm port.

**Figure 4:**
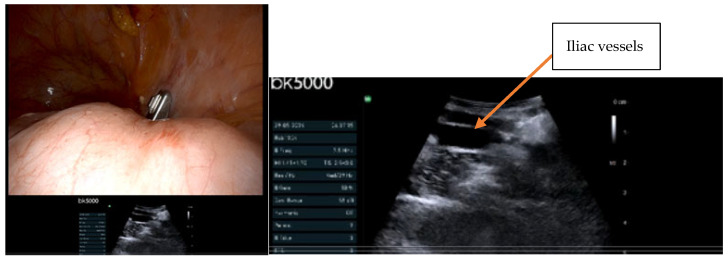
Identifying the right external iliac vessels under thick peritoneum using bk5000 (left image demonstrate tip of USS probe during the surgery, right image is the USS of the iliac vessels) (courtesy of A. Sayasneh).

**Figure 5:**
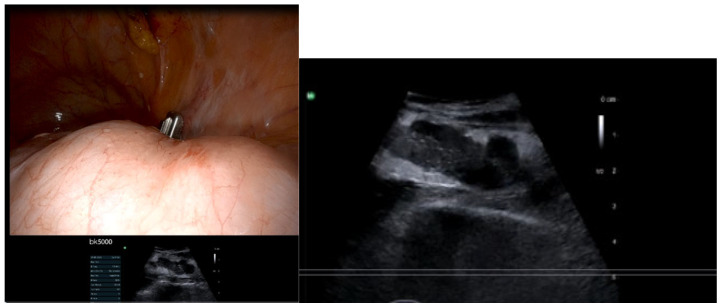
Identifying an enlarged lymph node. In the ultrasound scan image at the bottom of the picture, you can see the hypoechogenic large lymph node medial to the right external iliac vein (courtesy of A. Sayasneh).

**Figure 6:**
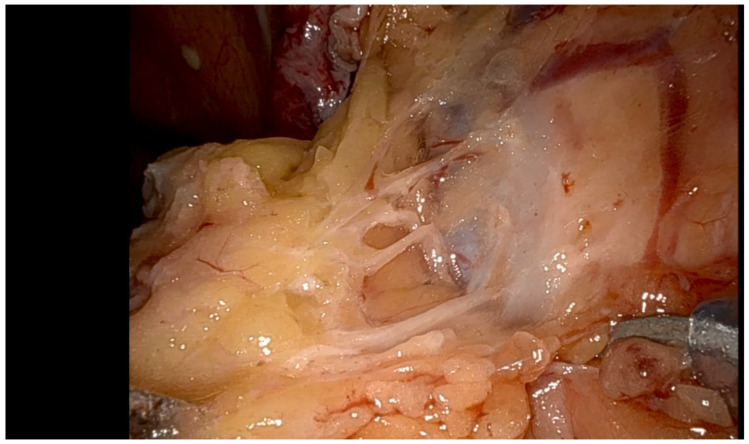
External LN with lymph ducts and right external vein. The lymph node targeted by ultrasound is removed and the right external iliac vein can be seen (courtesy of A. Sayasneh).

The integration of IOUS with advanced surgical platforms represents the next frontier in precision staging. Our institutional experience with the bk5000 ultrasound system during robotic procedures has demonstrated its capability to identify subcentimeter metastatic nodes in anatomically distorted fields (Figure 3, Figure 4, Figure 5 and Figure 6). This robotic–ultrasound synergy enables:

1. Real-time confirmation of suspicious nodes before excision
2. Precise needle guidance for targeted biopsy
3. Immediate assessment of resection completeness
4. Identification of critical vascular relationships to prevent injury

The clinical implementation of IOUS-guided nodal assessment demonstrates measurable improvements in surgical outcomes through enhanced intraoperative decision-making. Current evidence indicates that IOUS-based nodal evaluation reduces unnecessary lymphadenectomies in 30–40% of cases [12,26,30], with corresponding decreases in procedure-related morbidity including a 62% reduction in symptomatic lymphoceles, 58% lower incidence of chylous ascites, and 30% decreased rate of postoperative lymphedema. For patients with confirmed nodal metastases, IOUS facilitates complete oncologic resection of involved nodes while preserving unaffected lymphatic basins; an approach that maintains regional immune function and may potentially optimize the response to subsequent immunotherapy regimens. This selective nodal assessment paradigm represents a significant advancement in precision surgical oncology, where the balance between radical tumor excision and functional preservation is paramount. The technology’s ability to accurately differentiate reactive from malignant nodes translates into both reduced surgical morbidity and maintained therapeutic efficacy, addressing a critical need in contemporary gynecologic oncology practice.

The integration of intraoperative ultrasound (IOUS) into surgical practice aligns with the paradigm shift toward personalized therapeutic algorithms in gynecologic oncology. By providing real-time, reliable nodal assessment, IOUS addresses a critical surgical imperative: the accurate stratification of patients to optimize therapeutic intervention. Current clinical evidence positions this technology as a transformative tool for intraoperative decision-making, with the potential to redefine staging protocols.

Future technical refinements, including the incorporation of contrast-enhanced ultrasound and artificial intelligence-driven image analysis, may further enhance the diagnostic accuracy of IOUS. Such advancements could establish this modality as a reference standard for intraoperative nodal evaluation, potentially supplanting conventional approaches that lack comparable precision or immediate feedback. The ongoing evolution of IOUS technology reflects the broader movement in surgical oncology toward data-driven, individualized treatment strategies that balance oncologic efficacy with procedural morbidity.

- *Intraoperative Ultrasound in Cytoreductive Surgery: Advancing Optimal Tumor Debulking*

Peritoneal cancer deposits, also known as peritoneal carcinomatosis, represent a significant manifestation of transcoelomic spread in various malignancies. This condition is particularly common in gynecological cancers, especially ovarian cancer, where peritoneal and serosal deposits are often present at initial diagnosis [31]. Complete cytoreduction remains the cornerstone of surgical management in advanced epithelial ovarian cancer, where residual disease burden exhibits a well-characterized inverse relationship with progression-free and overall survival [32]. Mounting evidence from prospective trials and meta-analyses demonstrates that patients achieving complete gross resection (R0) experience median survival durations nearly double those with suboptimal debulking (1–2 cm residual disease), establishing maximal cytoreduction as the paramount surgical objective [33,34]. However, the technical challenges inherent to this endeavor are substantial, particularly when addressing metastatic deposits in anatomically complex regions such as the hepatoduodenal ligament, porta hepatis, and cardiophrenic lymph node (CPLN) basins [35].

The detection of peritoneal deposits relies on various imaging modalities. Ultrasound examination plays a valuable role in identifying suspicious features in ovarian masses, including peritoneal or omental deposits [36,37].

Multiple imaging techniques including CT, MRI, and PET/CT scans are employed for comprehensive evaluation to differentiate ovarian cancer from gastrointestinal tumors and predict chances of cytoreductive surgery [38]. Each modality offers distinct advantages [39]. However, the sensitivity of conventional imaging modalities is significantly influenced by lesion size. For peritoneal implants ≥ 0.5 cm, MRI, CT, and PET/CT demonstrate relatively good sensitivity at 95%, 84%, and 86%, respectively. However, when including implants < 0.5 cm, the sensitivity decreases dramatically to 40%, 38%, and 42%, respectively [40].

This size-dependent limitation is further highlighted in studies showing that PET/CT sensitivity for peritoneal metastases drops from an overall 72% to just 11% for nodules smaller than 5 mm [41].

IOUS addresses these limitations through real-time, high-resolution visualization of tumor deposits and their spatial relationships to critical anatomic structures. The clinical utility is particularly pivotal in three key domains of advanced cytoreduction:

### 3.1. Supradiaphragmatic/Cardiophrenic Lymph Node (CPLN) Disease Management

While the CPLN involvement is relatively uncommon, with some studies reporting an incidence of only 2.3%, their involvement is clinically significant as it typically indicates extensive disease spread [42].

The impact of CPLN resection on survival outcomes remains a subject of debate. While abnormally enlarged, unresected CPLNs may worsen survival in patients who have undergone complete intra-abdominal gross resection, several case series suggest that resection of enlarged paracardiac and cardiophrenic lymph nodes may prolong survival in carefully selected patients [43].

IOUS represents a valuable technique for the identification and precise localization of CPLN during surgical procedures [34]. The transdiaphragmatic ultrasound approach has been specifically documented for CPLN visualization. This technique involves using a convex contact probe through a transhepatic window to ultrasonographically identify enlarged CPLNs and precisely determine their location. Following the initial ultrasound scan and confirmation of the lymph node position, the diaphragm can be incised proximally to the lymphadenopathies, facilitating their removal. A subsequent intraoperative ultrasound control can then verify the absence of residual disease [13].

For optimal identification, surgeons can utilize a preoperative ultrasound to establish landmarks that serve as guides during the procedure. Key techniques include measuring the suspected lymph node’s size, determining its distance from the skin margin, and analyzing its shape, margins, and relationships with nearby anatomical structures. When these ultrasound parameters are confirmed intraoperatively, node identification becomes more efficient and effective [44].

### 3.2. Hepatic and Retroperitoneal Resection Guidance

**Figure 7:**
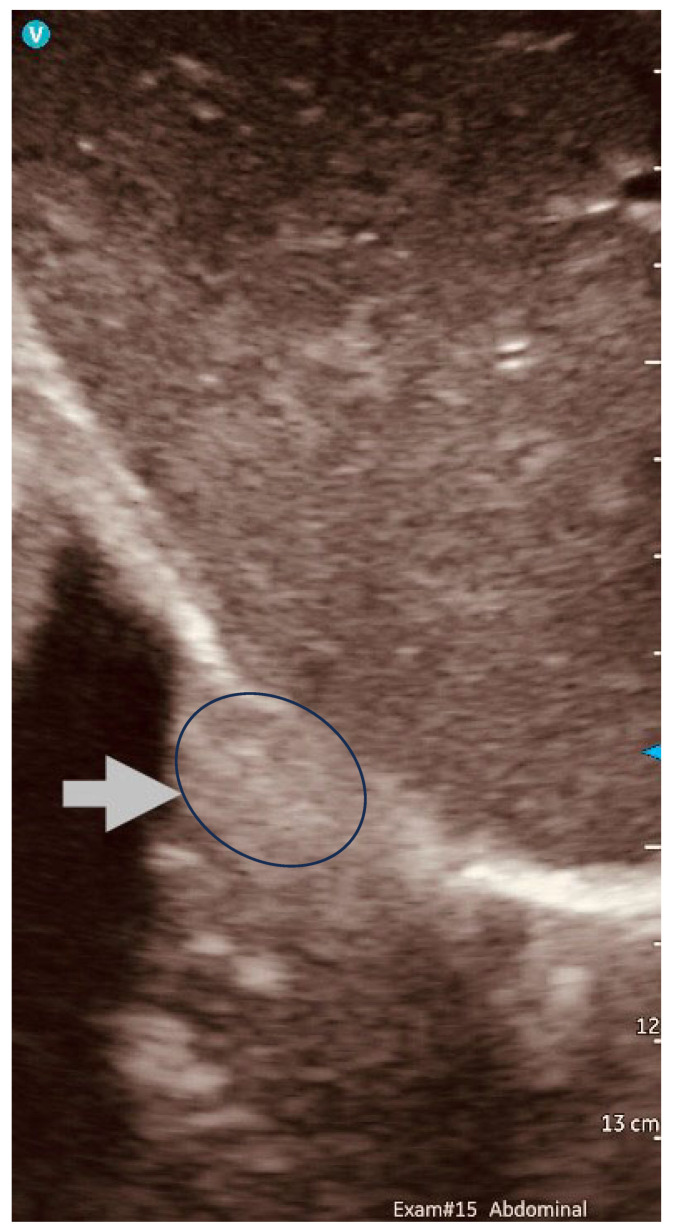
Introspective ultrasound scan image using a convex probe in open surgery to show a deep liver subcapsular deposit (arrow indicating deep liver deposit (circled)) (V on top-left is the orientation marker for GE Vuson indicating the ventral direction).

The complex anatomy of the hepatic hilum presents unique challenges for oncologic resection, where intraoperative ultrasound (IOUS) demonstrates significant utility in surgical planning and execution. Our institutional experience, unpublished data, with 47 consecutive upper abdominal cytoreductions, revealed that IOUS provides superior visualization of tumor–vascular relationships compared to conventional modalities, enabling the detection of subclinical hepatic metastases (mean diameter: 6.5 ± 2.1 mm) and precise delineation of tumor involvement within portal triad structures. Importantly, IOUS allows for real-time verification of resection margins, enhancing surgical precision. These capabilities led to intraoperative strategy modifications in 38% of cases, primarily through the identification of radiographically occult lesions. The frequent detection of subcentimeter metastases (Figure 7) suggests that current preoperative imaging may underestimate the extent of the disease in this anatomically complex region. These findings highlight the need for further investigation to quantify the survival impact of IOUS-detected occult lesions, standardize imaging protocols for hepatic hilar assessment, and evaluate cost-effectiveness relative to alternative staging approaches.

### 3.3. Precision Peritoneal Stripping

High-frequency transducers (7–15 MHz) enable differentiation between malignant implants and benign adhesions along peritoneal surfaces, particularly in the paracolic gutters and pelvic sidewalls [42]. This capability proves invaluable in recurrent disease settings, where fibrotic changes obscure conventional visual-tactile assessment. A recent prospective study [43] reported that IOUS-guided peritoneal resection reduced unnecessary radical procedures by 29% while increasing complete resection rates from 68% to 87% compared to conventional techniques.

IOUS significantly influences surgical decision-making by offering real-time and detailed anatomical insights that improve surgical precision especially when high-frequency transducers are utilized. In complex cancer surgery, IOUS has been shown to detect the exact extent of retroperitoneal and parenchymal hepatic tumor deposits and redefine the resection planes during operations, accommodating both oncological effectiveness and the preservation of vital liver parenchyma [45]. This approach allows for “radical but conservative surgery”, which is particularly beneficial in managing hepatocellular carcinoma and peritoneal cancers such as ovarian and colorectal with liver metastases, altering surgical strategies in approximately 30–35% of cases [46]. Furthermore, the ability of IOUS to identify and preserve crucial vascular structures also limits the need for major resections, ultimately minimizing post-surgery morbidity [47]. Additionally, IOUS assists in adapting the surgical plan intraoperatively by revealing residual tumors that may have gone undetected, thereby reducing the necessity for unwarranted aggressive surgical approaches [48].

IOUS precision could be further enhanced by integrating IOUS with additional imaging techniques. Notably, when IOUS is coupled with indocyanine green fluorescence imaging (ICG-FI), the combination has been shown to provide superior results. A study demonstrated that this merged approach could detect 29 lesions compared to just 15 lesions identified by IOUS alone. Moreover, the number of lesions detected using the combination was much higher than the nine lesions identified by preoperative CT scans alone [49].

**Figure 8:**
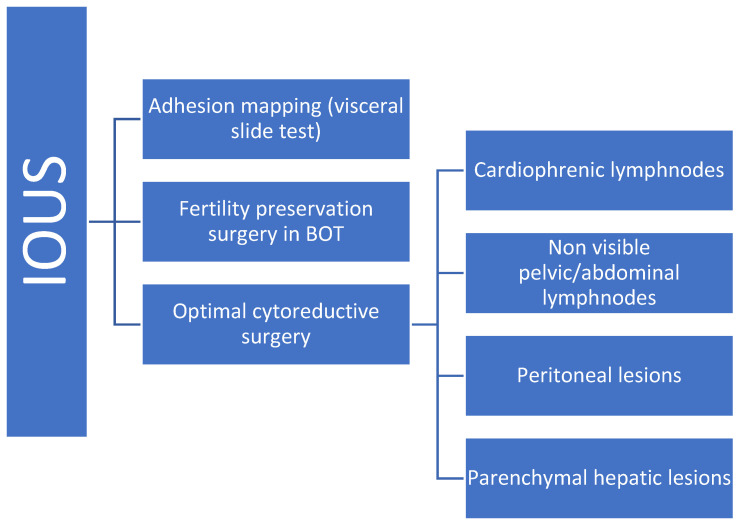
Illustration of IOUS in gynecological oncological surgery.

Figure 8 summarizes the different indications of IOUS in the literature.

## 4. Challenges and Limitations of Intraoperative Ultrasound in Gynecologic Oncology

While intraoperative ultrasound (IOUS) has emerged as a potentially transformative technology in gynecologic oncology surgery, its widespread clinical implementation faces several significant barriers that merit thorough examination. These limitations span technical, operational, educational, and economic domains, each presenting unique challenges that must be addressed to realize the full potential of this imaging modality.

### 4.1. Operator Dependency and the Learning Curve Challenge

The effectiveness of IOUS is fundamentally constrained by its operator-dependent nature, requiring a combination of technical expertise in both ultrasound physics and complex surgical anatomy [12].

The number of cases required to achieve competency in intraoperative ultrasound (IOUS) varies significantly depending on the specific application, prior ultrasound experience, and surgical expertise to understand the context. While in some cases competency in basic scanning can be achieved with as little as 5–10, complex scanning may require as many as 200 cases [50,51]. The learning curve is particularly steep for:

- Recognition of subtle sonographic features differentiating malignant from benign lesions
- Accurate correlation of two-dimensional ultrasound images with three-dimensional surgical anatomy
- Real-time integration of imaging findings into surgical decision-making

Even among experienced operators, significant interobserver variability persists in the interpretation of borderline findings, particularly for lesions < 1 cm or those with ambiguous vascular patterns [12]. This variability may lead to inconsistent surgical management decisions across institutions and individual practitioners.

### 4.2. Technical and Physical Limitations

The physics of ultrasound propagation impose inherent constraints on IOUS applications in gynecologic oncology. High-frequency transducers (7–15 MHz), while providing excellent spatial resolution for superficial structures, demonstrate markedly reduced penetration in patients with:

- Elevated body mass indices (BMI > 35)
- Extensive retroperitoneal disease
- Dense post-radiation fibrosis

The presence of surgical adhesions or excessive intra-abdominal fat can create significant acoustic shadowing and beam attenuation artifacts, potentially obscuring critical anatomical relationships [46]. The pathological changes associated with scarring—including fibrosis, fibrin deposition, and the proliferation of fibroblasts—create uneven tissue density that manifests as variations in brightness and gray values on ultrasound images [52,53]. This hyperechogenicity can obscure important anatomical structures and make it difficult to differentiate between healthy and pathological tissues.

Moreover, the technology remains limited in its ability to detect micro metastases (<2 mm) or characterize certain histologic subtypes (particularly desmoplastic or fibrotic nodal involvement), with false-negative rates approaching 12% in validation studies [30,34].

### 4.3. Standardization Deficit and Protocol Heterogeneity

Unlike established imaging modalities such as MRI or CT, which benefit from well-defined consensus guidelines, IOUS currently lacks universally accepted standards for:

1. Image acquisition parameters (frequency selection, depth adjustment, gain optimization)
2. Interpretation criteria for malignancy probability assessment
3. Procedural protocols for specific surgical scenarios (fertility-sparing resections, sentinel node mapping, etc.) [12].

This protocol heterogeneity creates significant disparities in clinical practice patterns between institutions. For example, while some comprehensive cancer centers employ rigorous multiparametric scoring systems for nodal assessment (incorporating size thresholds, cortical thickness measurements, and vascular patterning analysis), many institutions still rely on subjective morphological evaluation alone [12,26]. Such variability not only hampers multicenter research efforts but also complicates comparative effectiveness analyses.

### 4.4. Economic and Infrastructural Barriers

The financial implications of IOUS adoption present substantial obstacles, particularly for resource-constrained settings. The capital costs associated with high-end laparoscopic ultrasound systems typically exceed those of conventional laparoscopic equipment. Additional economic considerations include:

1. Significant maintenance costs of delicate transducers
2. Specialized sterilization requirements
3. Potential need for dedicated imaging personnel
4. Opportunity costs associated with prolonged operative times during the learning curve phase

In low-resource settings, these financial burdens may prove prohibitive, potentially exacerbating existing disparities in global cancer care delivery [49]. Even in well-resourced institutions, the cost-benefit calculus remains complex without robust prospective data demonstrating clear improvements in long-term oncologic outcomes.

Addressing these challenges will require a coordinated, multidisciplinary approach encompassing:

1. Development of structured training curricula incorporating virtual reality simulation and competency-based progression
2. Establishment of evidence-based consensus guidelines through professional society collaborations
3. Technological innovations in probe design (including 3D matrix arrays and elastography capabilities)
4. Health economic analyses to quantify the long-term value proposition [4,12,44]

While the diagnostic and therapeutic potential of IOUS in gynecologic oncology is undeniable, its successful integration into routine practice demands clear-eyed recognition of these limitations. The path forward requires balanced investment in technological refinement, education standardization, and outcomes research to transform IOUS from a promising innovation into a reliably effective component of precision cancer surgery. Only through such comprehensive efforts can this technology achieve its potential to improve surgical outcomes while maintaining cost-effectiveness across diverse healthcare settings.

## 5. Future Perspectives: Advancing Intraoperative Ultrasound in Gynecologic Oncology Through Innovation and Standardization

The evolution of intraoperative ultrasound (IOUS) in gynecologic oncology stands at a critical juncture, where technological innovation, educational reform, and protocol standardization must converge to realize its full potential. As we look ahead, several key priorities emerge that will shape the next decade of progress in this transformative surgical adjunct.

### 5.1. Standardization of Imaging Protocols and Interpretation Criteria

The current heterogeneity in IOUS application represents one of the most pressing challenges to its widespread adoption. The development of evidence-based, procedure-specific guidelines—endorsed by major gynecologic oncology societies—will be essential to establish uniformity in:

- Malignancy risk stratification of indeterminate lesions
- Criteria for fertility-sparing resection margins
- Standardized reporting terminology for intraoperative findings
- Integration with sentinel lymph node algorithms

Such standardization will not only improve reproducibility across institutions but also facilitate meaningful multicenter research collaborations. The creation of an international IOUS registry could accelerate this process, pooling data from high-volume centers to identify best practices and establish predictive models for clinical decision-making.

### 5.2. Technological Innovations on the Horizon

Next-generation ultrasound technologies promise to overcome many current limitations. Three-dimensional ultrasound reconstruction is poised to revolutionize spatial understanding of tumor geometry, particularly for complex ovarian masses. Contrast-enhanced ultrasound (CEUS), with its ability to characterize microvascular patterns in real time, may provide intraoperative “virtual histology” to guide resection boundaries. Most transformative may be the integration of artificial intelligence, where deep learning algorithms trained on vast ultrasound datasets could provide:

- Automated lesion detection with quantified probability scores
- Real-time differentiation between benign and malignant features
- Predictive analytics for occult metastasis risk

The miniaturization of high-frequency probes for robotic platforms and the development of “smart” laparoscopic instruments with embedded ultrasound capabilities will further enhance surgical precision in minimally invasive approaches.

### 5.3. Educational Imperatives for the Next Generation

Bridging the expertise gap requires a fundamental restructuring of surgical training paradigms. Simulation-based mastery learning programs—incorporating virtual reality platforms with haptic feedback—should become a prerequisite before live-patient application. High-fidelity models that replicate both normal anatomy and pathologic findings (e.g., deep infiltrating endometriosis, ovarian tumor pseudocapsules) will be crucial for skill acquisition.

The establishment of centralized “centers of excellence” for IOUS training, coupled with telemedicine platforms allowing real-time proctoring, could democratize access to expertise. Incorporating ultrasound certification into gynecologic oncology fellowship requirements would ensure competency across the specialty.

### 5.4. Health Systems Integration and Value Assessment

Widespread adoption will depend on demonstrating not just clinical efficacy but also healthcare value. Prospective cost-effectiveness analyses must quantify how IOUS reduces operative time, decreases unnecessary lymphadenectomies, and minimizes reoperation rates. Device manufacturers must address the current economic barriers through innovative financing models that make advanced probes accessible across resource settings.

### 5.5. Telemedicine Integration and Virtual Consultation Models

The rapid evolution of telemedicine presents transformative opportunities for enhancing IOUS utilization in gynecologic oncology. Emerging platforms enabling real-time virtual consultation could allow expert sonographers to guide IOUS interpretation remotely during complex procedures, effectively democratizing access to specialized expertise. This “tele-IOUS” model would be particularly valuable for: (1) community hospitals lacking on-site ultrasound specialists, (2) training programs requiring real-time proctoring, and (3) international collaborative surgeries where second-opinion consultation is needed.

Technological infrastructure for such integration already exists through secure, HIPAA-compliant platforms capable of streaming high-definition ultrasound images with sub-second latency. Future developments may incorporate augmented reality overlays to highlight suspicious areas directly in the surgeon’s visual field, coupled with artificial intelligence algorithms that provide predictive analytics during the consultation. However, implementation will require addressing regulatory considerations (licensing across jurisdictions), establishing quality control standards for remote interpretation, and developing reimbursement models for virtual intraoperative consultations. The convergence of 5G networks, cloud-based image processing, and miniaturized ultrasound hardware suggests that tele-IOUS could become a routine component of precision surgery within the next decade.

## 6. Conclusions

IOUS is emerging as a pivotal innovative tool in gynecologic oncology, with the potential to establish new standards for surgical precision. By providing real-time, patient-specific anatomic data, this technology overcomes critical limitations inherent in both preoperative imaging modalities and conventional intraoperative visualization techniques. While implementation challenges remain, including the need for standardized protocols, specialized training, and equitable technology access, the accumulating evidence strongly supports IOUS’s transition from an optional tool to an essential component of contemporary oncologic surgery.

Current prospective trials and technological advancements suggest that IOUS-guided resection may soon become a quality benchmark in gynecologic cancer surgery. This evolution represents more than a technical advancement; it heralds a fundamental shift from macroscopic surgical approaches to an era of microscopic precision. In this new paradigm, real-time imaging informs each surgical decision, resection margins are objectively optimized, and operative strategies are continuously refined through the integration of advanced technology with surgical expertise.

The clinical implications of this transformation are profound. As the field progresses, we anticipate demonstrable improvements in both oncologic outcomes and quality of life for patients. By enabling more accurate staging, more complete tumor resection, and reduced surgical morbidity, IOUS exemplifies the potential of technology-enhanced precision surgery to advance the standard of care in gynecologic oncology.

## Data Availability

Not applicable.
